# Preoperative Skin Preconditioning with Autologous PRP and SVF: Effects on Cutaneous and Adnexal Repair in a Rabbit Pilot Study

**DOI:** 10.3390/biomedicines14102399

**Published:** 2026-10-20

**Authors:** Meirambek Askarov, Ulpan Batenova, Ilona Pak, Elyarbek Tashmetov, Dmitriy Klyuyev, Min Sung Tak, Yevgeniy Kamyshanskiy

**Affiliations:** 1 Department of Surgical Diseases, Karaganda Medical University, Karaganda 100012, Kazakhstan; meiramaskarov@mail.ru (M.A.); dr.park@bk.ru (I.P.); 2 Institute of Life Sciences, Karaganda Medical University, Karaganda 100008, Kazakhstan; batenovaulpan@gmail.com (U.B.); klyuev@qmu.kz (D.K.); 3 Plastic and Reconstructive Surgery Department, Soonchunhyang University Hospital, Seoul 04401, Republic of Korea; tarkms@naver.com; 4 Pathology Department, Karaganda Medical University, Karaganda 100008, Kazakhstan; kamyshanskiy@qmu.kz

**Keywords:** wound healing, platelet-rich plasma, stromal vascular fraction, preoperative regenerative medicine, rabbit model, contraction indices

## Abstract

**Background:** Optimal healing of surgical skin wounds remains a critical issue in modern surgery. This study evaluated the effect of preoperative administration (before wound creation) of platelet-rich plasma (PRP) and stromal vascular fraction (SVF) on wound healing in a rabbit full-thickness excisional skin wound model. **Methods:** Forty animals were randomized into five groups: control (no treatment), 0.9% NaCl, SVF, PRP, and PRP + SVF. Biological agents were injected intradermally 24 h before standardized 2-cm excisional wounds were created. Histological, morphometric, IHC and contraction indices (SCI, DCI, WCI) were assessed at days 7, 14, and 30. **Results:** Preoperative administration of PRP + SVF significantly enhanced cutaneous wound repair, with early re-epithelialization evident by day 7 and complete epithelial restoration by day 14 compared with the control group. Histomorphometric analysis revealed that the PRP + SVF group demonstrated the highest angiogenic response on day 7 (median 24 vs. 12 in the control group, *p* = 0.027), the greatest regenerative tissue thickness on day 14 (84.5% vs. 62.5%, *p* = 0.021), and the highest extracellular matrix remodeling index on day 30 (0.655 vs. 0.404), accompanied by regeneration of skin appendages (median score 3 vs. 1 in the control group, *p* = 0.005). **Conclusions:** These findings demonstrate that preoperative administration of PRP combined with SVF improves the histological and histomorphometric outcomes of cutaneous wound healing. These preliminary findings provide a rationale for further experimental studies to validate and optimize this approach, to elucidate the underlying mechanisms, and to clarify the specific contribution of preoperative tissue conditioning.

## 1. Introduction

Optimal healing of surgical skin wounds remains a critical issue in modern surgery and dermatologic oncology. Surgical excision of skin lesions inevitably produces tissue defects, and impaired healing may result in delayed epithelialization, infection, fibrosis, or unfavorable scar formation [1,2]. Therefore, development of effective regenerative strategies aimed at improving wound repair remains an important clinical objective.

Platelet-rich plasma (PRP) has been extensively investigated as an autologous biologic therapy due to its high concentration of growth factors, which regulate angiogenesis, fibroblast proliferation, and extracellular matrix synthesis, thereby promoting wound healing and tissue regeneration [3,4,5]. Similarly, the stromal vascular fraction (SVF) derived from adipose tissue contains mesenchymal stromal cells, endothelial progenitors, and immunomodulatory populations capable of enhancing vascularization, modulating inflammation, and promoting tissue repair [6,7]. Although both therapies have demonstrated significant regenerative potential, existing experimental and clinical studies have almost exclusively focused on their administration after tissue injury, while their preoperative use to biologically precondition the future wound bed remains largely unexplored.

Accumulating evidence suggests that preoperative tissue conditioning may enhance postoperative tissue repair by activating regenerative pathways before surgical injury occurs [8,9,10,11]. However, this concept has been investigated primarily using physical preconditioning approaches, whereas the potential of biological preconditioning with PRP and SVF prior to tissue injury remains largely unexplored. It is currently unknown whether preoperative administration of these biologic products can establish a more favorable regenerative microenvironment, thereby improving the subsequent healing response following surgical wounding.

To the best of our knowledge, no experimental studies have evaluated the combined local preoperative administration of PRP and SVF before the creation of a full-thickness excisional skin wound. Therefore, the aim of the present study was to determine whether local intradermal administration of PRP combined with SVF 24 h before surgery enhances wound healing in a rabbit model of full-thickness excisional skin injury.

## 2. Materials and Methods

### 2.1. Ethical Oversight and Animal Husbandry

The experimental protocol was conducted at the Animal Laboratory and Pathology Unit of the Karaganda Medical University (Karaganda, Kazakhstan). Institutional approval was secured from the University Animal Care Committee (UACC) (Protocol No. 21, 10 December 2024). All animal care and surgical procedures were performed in strict compliance with the Guide for the Care and Use of Laboratory Animals (8th edition).

A total of 40 male outbred rabbits (4–5 months old; body weight 2.5–3.5 kg) were utilized. Prior to experimental induction, animals underwent a 14-day acclimatization period in individual stainless-steel cages. Environmental conditions were maintained at a constant temperature of 22 ± 2 °C and 40–50% relative humidity under a 12-h light/dark cycle. Rabbits were provided with a standardized pelleted diet and tap water ad libitum.

The present study has a pilot character and was designed as a preliminary evaluation of the effectiveness of different therapeutic approaches to skin wound healing in accordance. No a priori sample size calculation was performed. The experiments were conducted in compliance with EU Directive 2010/63/EU on the protection of animals used for scientific purposes, and at the end of the study the rabbits were transferred to a teaching farm instead of being euthanized.

### 2.2. Experimental Design and Randomization

**Figure 1:**
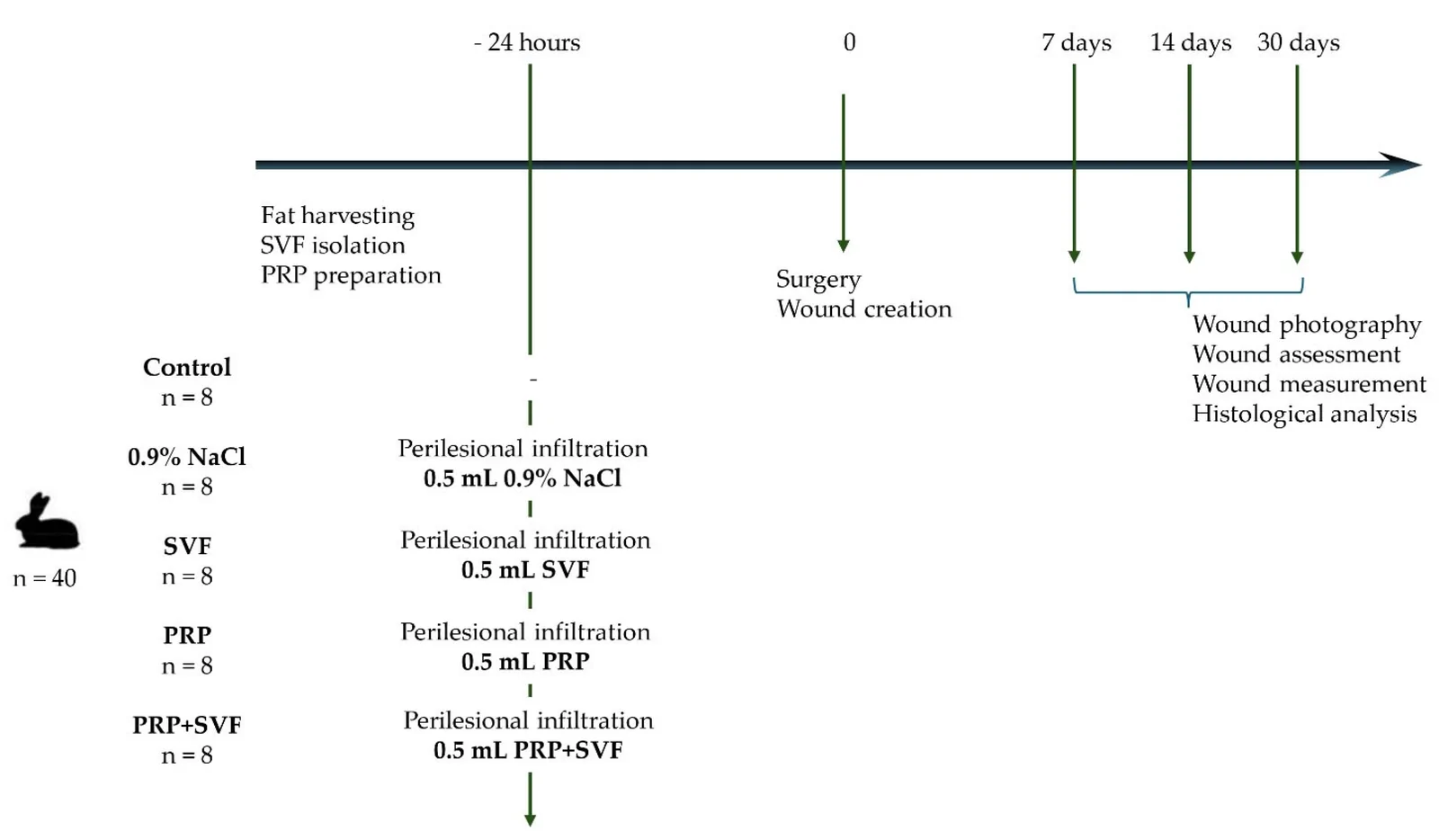
Schematic representation of the study design time points.

The experimental design is illustrated in Figure 1.

The sample size was determined in accordance with the 3Rs principles (Replacement, Reduction, and Refinement) proposed by Russell and Burch [12,13].

Forty male outbred rabbits were randomly assigned to five equal experimental groups (n = 8 per group) using a computer-generated randomization sequence:

- Group 1 (Control): no treatment administered.
- Group 2 (0.9% NaCl): administration of 0.5 mL of 0.9% NaCl solution.
- Group 3 (SVF): administration of 0.5 mL of stromal vascular fraction.
- Group 4 (PRP): administration of 0.5 mL of platelet-rich plasma.
- Group 5 (PRP + SVF): administration of a combination of PRP and SVF in a 1:1 ratio (0.25 mL of each; total volume 0.5 mL).

Preoperative treatment was carried out 24 h before surgery by perifocal intradermal injection into the area of the planned wound. The 24-h interval was selected to permit early activation of PRP- and SVF-mediated regenerative pathways before surgical injury while minimizing pre-existing structural remodeling of the target tissue.

The total volume (0.5 mL) was evenly distributed among 4–6 intradermal injection points along the wound perimeter. Four standardized 2-cm full-thickness excisional wounds were created on the dorsal skin of each animal. Wound healing was assessed on days 7, 14, and 30. At each time point, one wound per animal was randomly selected (n = 8 in each group) and excised for histological and morphometric analysis. To ensure uniformity of tissue sampling, specimens were collected from predefined areas located at an equal distance from the original wound margins (day 0), using previously applied reference markings. Each animal (n = 8 in each group) was considered the primary unit of analysis for all statistical comparisons.

### 2.3. Preparation of Biological Products

#### 2.3.1. Platelet-Rich Plasma (PRP) Isolation

The double-centrifugation technique was employed to obtain leukocyte-rich platelet-rich plasma (L-PRP), with intentional collection of the buffy coat to include both platelets and concentrated leukocytes, thereby enhancing the initial inflammatory phase of combined preoperative biological conditioning. Autologous whole blood (3.5 mL) was collected from the marginal auricular vein into 4.5 mL tubes containing 3.8% sodium citrate (Guangzhou Improve Medical Instruments Co., Ltd., Guangzhou, China) as an anticoagulant.

1. First Step: Initial centrifugation was performed using a bench-top centrifuge (OPn-3.01 “Dastan”) at 450× *g* (2400 rpm) for 10 min.
2. Second Step: The supernatant plasma layer, including the buffy coat (leukocyte layer), was carefully harvested and subjected to a second centrifugation at 850× *g* (3600 rpm) for 15 min.

The upper plasma fraction was aspirated until approximately 2 mL of residual plasma remained at the bottom of the tube. For PRP preparation, the baseline platelet count in the rabbit peripheral blood was determined (284 ± 42 × 10^9^/L). The final PRP concentration was standardized to 1.2 × 10^9^ cells/mL, representing approximately a 4.2-fold increase over baseline, ensuring therapeutic potency [14,15,16]. Before administration, PRP was exposed to a single freeze–thaw cycle at −20 °C to induce platelet degranulation and maximize the release of bioactive growth factors contained within platelet α-granules. PRP was prepared individually for each animal from autologous blood samples and used immediately after thawing.

#### 2.3.2. Stromal Vascular Fraction (SVF) Extraction

Adipose tissue was harvested from the inguinal region using the Coleman technique. Lipoaspirate was collected via manual aspiration using a universal liposuction cannula and 10 mL Luer-Lok syringes (Tulip Medical Products, San Diego, CA, USA). The mean tissue mass collected was approximately 3 g. The SVF was extracted through mechanical emulsification without enzymatic digestion:

- The lipoaspirate was sequentially passed through 2.4 mm and 1.2 mm filters (Nanofat Filter Transfer Set, Tulip Medical Products, San Diego, CA, USA).
- To obtain the stromal vascular fraction (SVF) via mechanical emulsification, the lipoaspirate was transferred between two 10-mL syringes connected by a Luer-to-Luer transfer adapter (Tulip Medical Products, San Diego, CA, USA). To ensure consistent fragmentation, 30 passes were performed through a 2.4-mm aperture, followed by an additional 30 passes through a 1.2-mm aperture until a homogenous, creamy consistency was achieved. No enzymatic digestion was used.
- Further refinement was achieved using a disposable 600/400 µm filter device Nanofat Filter Transfer Set, Tulip Medical Products, San Diego, CA, USA).
- The resulting suspension was centrifuged at 1500 rpm for 5 min. The supernatant and the oil layer (released from disrupted mature adipocytes) were discarded.

The viability of the obtained SVF cells was assessed using the Trypan Blue exclusion test, yielding an average viability of 92.4 ± 3.1%. Flow cytometry (Partec Cyflow Space, Sysmex Partec GmbH, Görlitz, Germany) confirmed the cellular composition, identifying a CD34+/CD45− population consistent with adipose-derived regenerative cells. For final application, 50,000 SVF cells (derived from ~2 g of adipose tissue) were diluted in 0.5 mL of isotonic saline solution (0.9% NaCl) and stored at 4 °C until use [17,18].

#### 2.3.3. Preparation of the PRP–SVF Combination

**Figure 2:**
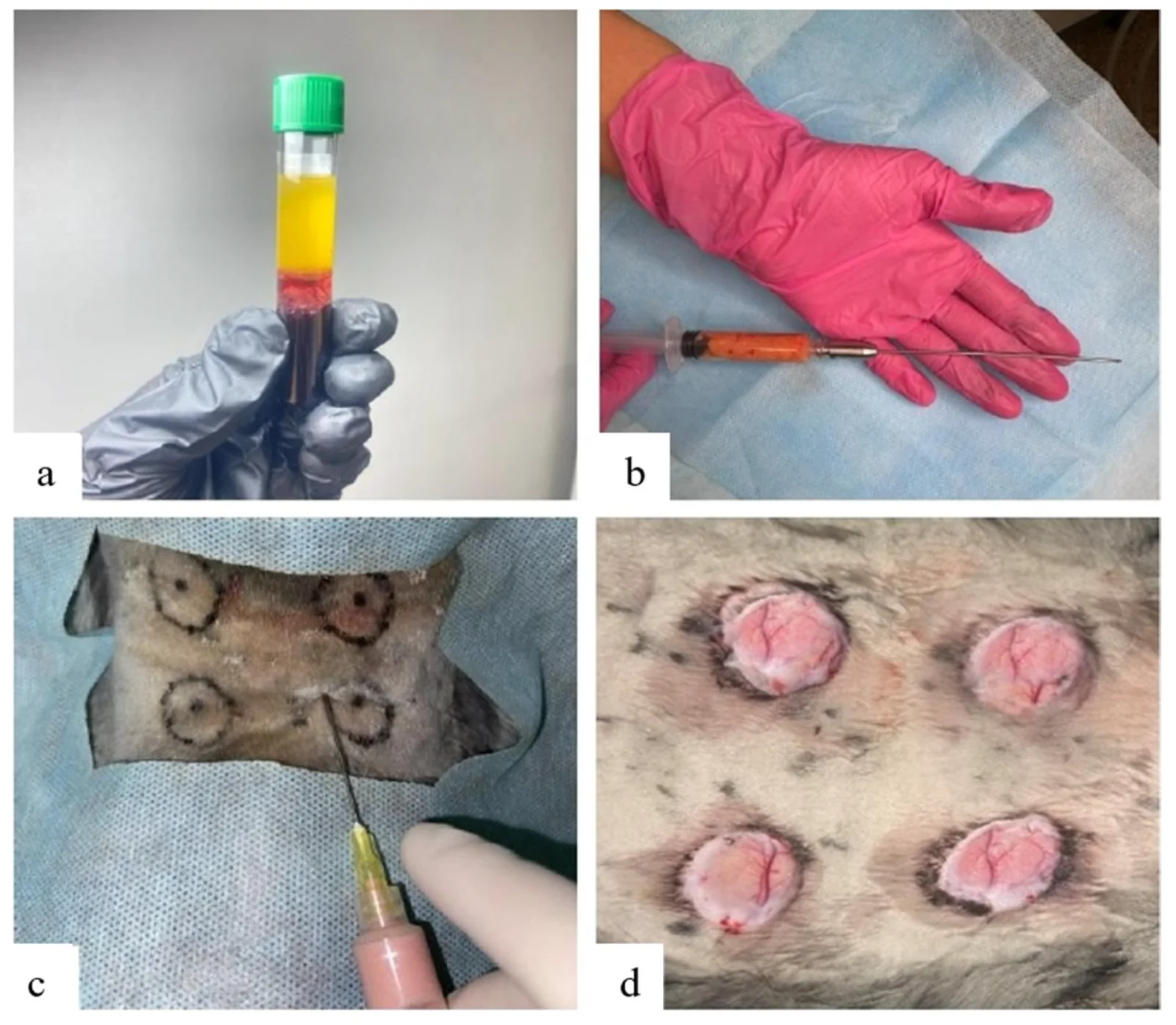
Preparation and perilesional infiltration of platelet-rich plasma (PRP) and stromal vascular fraction (SVF). (**a**) PRP obtained after centrifugation; (**b**) lipoaspirate collection for SVF isolation; (**c**) perilesional infiltration of PRP + SVF; (**d**) macroscopic appearance of the experimental wounds (PRP + SVF). The images are for illustrative purposes only, are provided to demonstrate the standardized methodology, and do not contain results obtained in the present study.

For Group 5, equal volumes of PRP (0.25 mL) and SVF (0.25 mL) were transferred into a sterile Luer-Lok syringe (Becton, Dickinson and Company, Franklin Lakes, NJ, USA). Homogenization was ensured by passing the suspension through sterile titanium NanoFat transfer Luer-to-Luer filters (Tulip Medical Products, San Diego, CA, USA), facilitating a uniform distribution of platelets and stromal cells. Preparation and perilesional infiltration of platelet-rich plasma (PRP) and stromal vascular fraction (SVF) are illustrated in Figure 2.

### 2.4. Surgical Induction of the Wound Model

All surgeries were performed by a single operator to minimize technical variability. General anesthesia was induced via intramuscular injection of a tiletamine/zolazepam (20 mg/kg) and xylazine (3 mg/kg) cocktail, supplemented with oxygen [19]. After hair removal and antiseptic skin preparation with povidone–iodine, the dorsal surface of each animal was marked using a custom-made sterile template (2 cm in diameter, prepared in-house at Karaganda Medical University, Karaganda, Kazakhstan), which served as a guide for standardizing the location and size of the created wound defects. This marking was also used to account for the degree of skin contraction during healing when biopsy specimens were collected.

Four circular wounds (2 cm diameter) were created in the paravertebral subcostal region, maintaining a minimum of 2 cm between defects. The excision involved the epidermis and dermis down to the subcutaneous layer, with partial involvement of the underlying adipose tissue. Hemostasis was achieved via sterile gauze compression.

Postoperative Care:

- Analgesia: Ketoprofen 10% (3 mg/kg, s.c.) once daily for 3 days.
- Wound Management: Daily irrigation with 0.05% chlorhexidine and povidone–iodine; wounds were left uncovered.
- Systemic Monitoring: Weekly measurement of body weight and rectal temperature.

### 2.5. Gross Examination

Digital photographs of the wounds were taken to monitor the regeneration process. Wound healing was photographed by an investigator blinded to group allocation. Macroscopic wound assessment, planimetric measurements, and calculation of wound contraction indices were performed using coded digital images by investigators blinded to the allocation of animals to the experimental groups.

The wounds were photographed on days 0, 7, 14, and 30 after wound creation, using a ruler for measurement and assessment of the healing process.

The degree of wound closure was calculated by measuring the largest diameter (initial size) and the smallest diameter (open wound area) of the wound and expressing healing as a percentage according to the following formula: (largest diameter − smallest diameter)/largest diameter) × 100 [20].

Macroscopic evaluation of each wound at different study time points was performed using the Oppenheimer scale [21].

### 2.6. Histological Processing

#### 2.6.1. Tissue Processing

Full-thickness tissue specimens were excised together with a 2–3 mm margin of adjacent intact skin to a depth of approximately 4–5 mm. Samples were gently flattened on a cork surface to prevent tissue distortion and immediately fixed in 10% neutral buffered formalin (ErgoProduction LLC, Saint Petersburg, Russia) at 4 °C for 24 h.

Each specimen was assigned a coded identifier indicating the animal number. Histological evaluation and histomorphometric measurements were performed on coded tissue sections by investigators blinded to the allocation of animals to the experimental groups.

Following fixation, tissues were rinsed in running tap water, dehydrated in graded ethanol, cleared in xylene, and embedded in paraffin. Serial sections (3–4 μm thick) were prepared using a rotary microtome (YD-315, Jinhua YIDI Medical Appliance Co., Ltd., Jinhua, China) and mounted on glass slides (Bio-Optica, Milan, Italy).

After deparaffinization and rehydration, sections were stained with hematoxylin and eosin (H&E), Masson’s trichrome, and orcein. Slides were mounted with coverslips using a permanent mounting medium and air-dried (Labiko LLC, Saint Petersburg, Russia). Histological evaluation was performed using a Zeiss AxioLab 4.0 microscope (Carl Zeiss Microscopy GmbH, Jena, Germany).

#### 2.6.2. Hematoxylin and Eosin Staining

For H&E staining, sections were immersed in Mayer’s hematoxylin for 15 min, rinsed under running water for 5 min, and counterstained with eosin for 1 min. This staining protocol was used for assessment of general tissue morphology, epithelial architecture, and inflammatory cell infiltration.

#### 2.6.3. Masson’s Trichrome Staining

Masson’s trichrome staining was performed using a commercially available kit (Trichrome Stain [Masson], ErgoProduction LLC, Saint Petersburg, Russia) according to the manufacturer’s instructions. This method was used to evaluate collagen deposition and connective tissue organization. Mature type I collagen fibers were visualized as dense dark blue structures.

### 2.7. Immunohistochemical Analysis

Additional sections were immunostained with a monoclonal antibody against ERG (clone EPR3864; VENTANA Medical Systems, Inc., Tucson, AZ, USA) using an automated BOND staining system (Leica Biosystems, Melbourne, Australia). ERG-positive microvessels were identified by nuclear staining of endothelial cells. Staining specificity was assessed using appropriate positive and negative controls.

For microvessel density analysis, sections were initially examined at low magnification (×40) to identify areas with the highest density of stained microvessels. Five non-overlapping fields with the highest vascular density were selected for counting at ×200 magnification (0.950 mm^2^ per field). A cluster of endothelial cells with brown-stained nuclei, clearly separated from adjacent microvessels, was counted as a single microvessel. The mean microvessel density across the five selected fields was considered the microvessel density for each specimen.

### 2.8. Histomorphometric and Semi-Quantitative Analysis

#### 2.8.1. Semi-Quantitative Scoring

Histological features were evaluated using a semi-quantitative 4-point scale (0–3) according to previously described criteria [22,23,24]. The scoring criteria for each parameter are presented in Table 1.

Restoration of epithelial continuity was defined as the presence of a continuous epithelial layer covering the wound surface.

#### 2.8.2. Dermal/Granulation Tissue Thickness

Dermal or granulation tissue thickness was measured as the perpendicular distance from the basal membrane of the epidermis to the interface with the underlying muscle or subcutaneous adipose tissue. Thickness was expressed as a percentage relative to intact skin from the same animal. Five measurements were obtained per wound section, and the mean value was calculated for each specimen. Areas affected by fixation artifacts (e.g., tissue folding or mechanical damage) were excluded from analysis.

#### 2.8.3. Wound Contraction Indices

Wound contraction was assessed using the indices described by Lemo N [25]. Matrix remodeling was assessed using the modified Matrix Remodeling Index (MMRI), with day 7 taken as the baseline time point.

### 2.9. Statistical Analysis

The individual animal (n = 8 per group) was defined as the primary unit of analysis. Data distribution was assessed using the Shapiro–Wilk test. Since the data followed a non-normal distribution, the Kruskal–Wallis test was employed for multi-group comparisons, followed by Dunn’s post hoc test with Bonferroni correction for pairwise differences. Quantitative results are expressed as Median and Interquartile Range (IQR: 25th–75th percentiles). All analyses were conducted using SPSS 20.0 (IBM Corp., Armonk, NY, USA) and STATISTICA 10.

## 3. Results

### 3.1. Macroscopic Findings

Macroscopic evaluation showed that in all groups the wounds healed without signs of infection. At the initial stage, the wound defects were characterized by irregular margins and a depressed crater-like wound bed with exposure of the underlying tissues. On day 7, in all groups, the color of the wound surface varied from pale pink to dark red, and the wound bed was covered with granulation tissue with areas of drying and dry crust formation. No necrosis or marked exudate was observed.

On day 14, in the control, 0.9% NaCl, and SVF groups, uneven coverage of the wound bed with granulation tissue was noted, along with dense crusts and scar formation with a heterogeneous structure and moderate hyperemia. In the PRP and PRP + SVF groups, signs of active crust detachment with exposure of newly formed pink skin were observed.

On day 30, in the control and 0.9% NaCl groups, the wounds were covered at the margins by scars with areas of pigmentation and hyperemia, while dense crusts remained in the central wound zone. In the SVF and PRP groups, dense grayish-red scars with areas of dry grayish-brown crusts had formed. In the PRP + SVF group, complete wound closure with formation of a thin grayish-white scar was observed.

**Figure 3:**
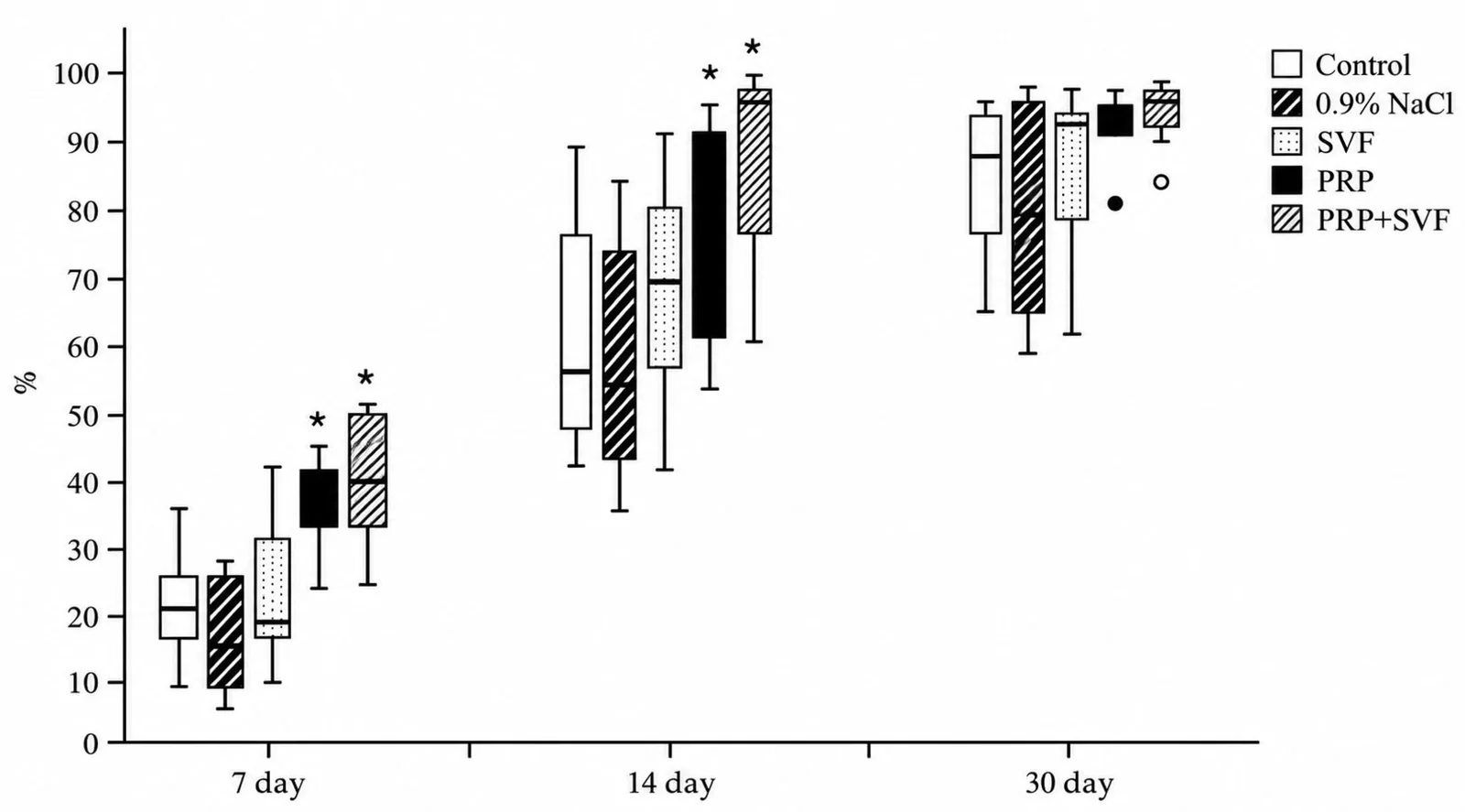
Macroscopic assessment of wound healing in the study groups (% wound closure at each time point). Statistical analysis performed via Kruskal–Wallis test followed by Dunn’s post hoc with Bonferroni correction. * Bonferroni-adjusted *p* < 0.05 vs. Control.

The planimetric assessment of wound contraction in the study groups is presented in Figure 3.

### 3.2. Histological Examination

#### 3.2.1. Histopathological and Morphometric Evaluation of Wound Healing on Days 7, 14, and 30

**Figure 4:**
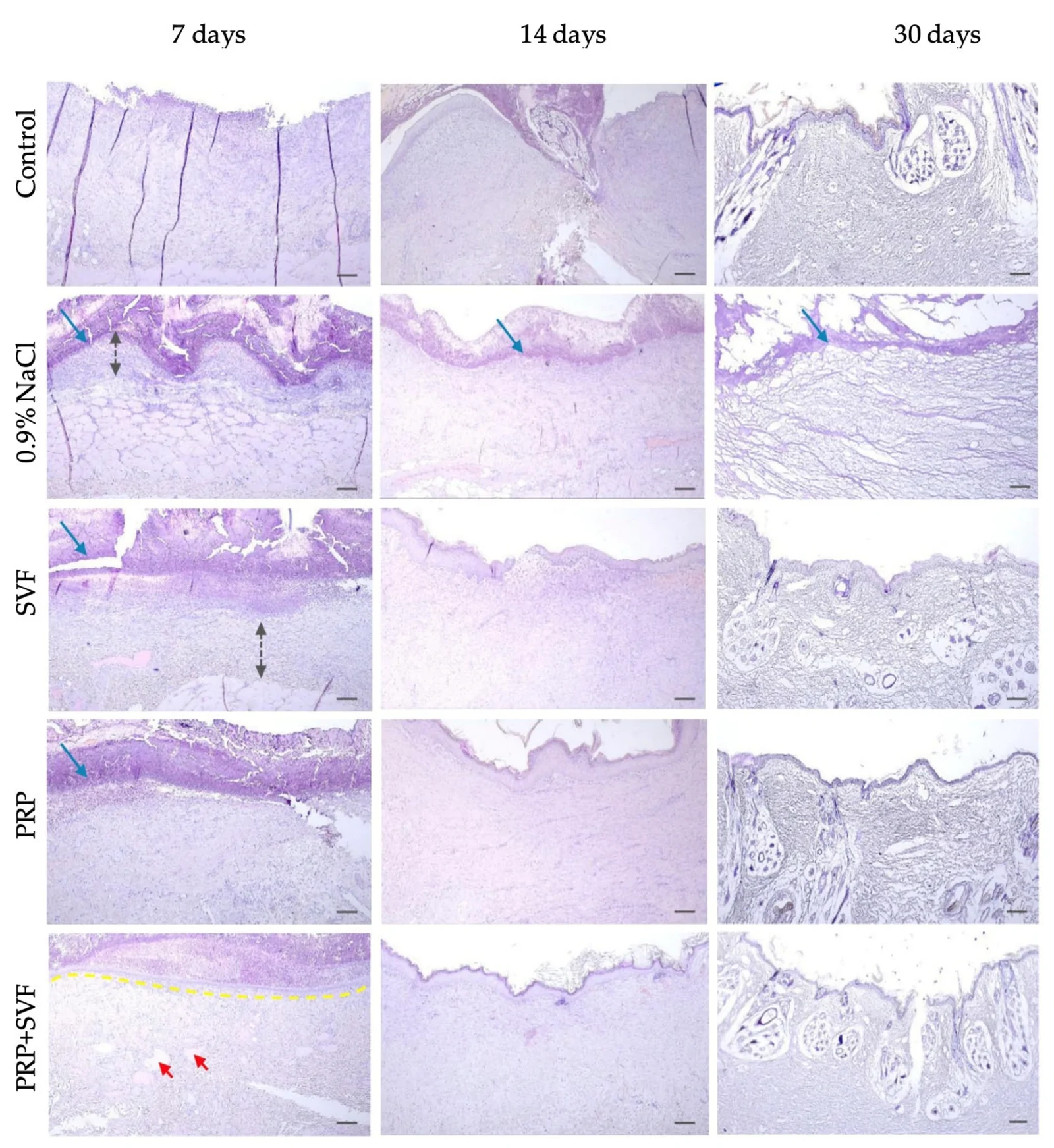
Hematoxylin and eosin-stained sections of healing wounds on day 7, 14, 30. Representative microphotographs of wound biopsies from each treatment group were obtained near the center of the injured area. The epithelium (yellow dash line), newly formed blood vessels (red arrows), and scab (blue arrows) are clearly identifiable.

**Figure 5:**
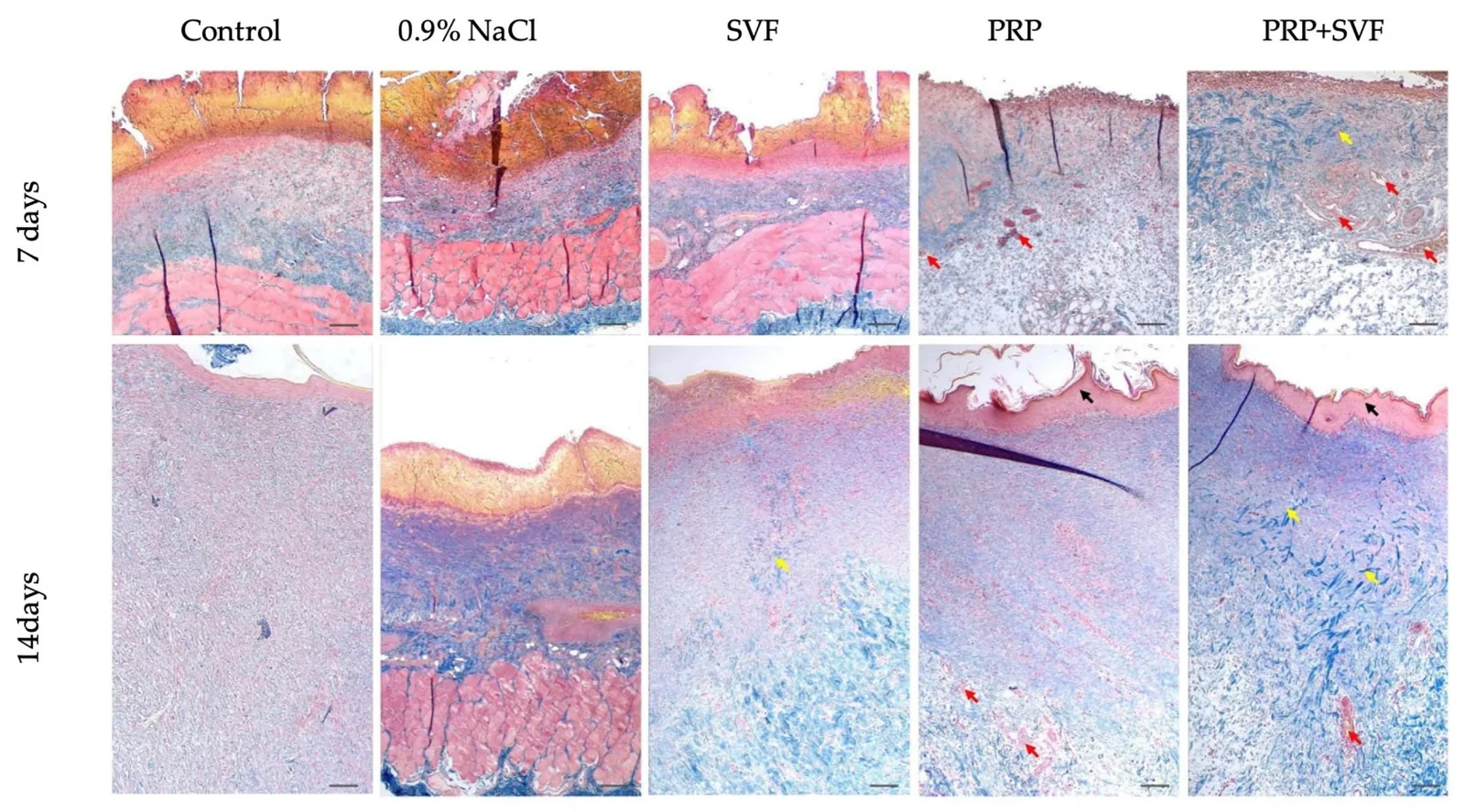
Masson’s 60 trichrome-stained sections of healing wounds at 7 days and 14 days. All micrographs are representative images of wound biopsies in each treatment group, taken near the center of the injured area. Histochemical staining of collagen proteins with Masson’s trichrome, 100× magnification, scale bar 500 μm.

The histopathological and morphometric evaluation of wound healing on days 7, 14, and 30 is presented in Supplementary Materials (Table S1) and Figure 4 and Figure 5.

Day 7: Control, 0.9% NaCl, SVF, and PRP groups: the surface of the wound defect is covered with a fibrinonecrotic scab (blue arrows). PRP + SVF group: the wound surface is completely re-epithelialized (yellow dashed line), and newly formed blood vessels are identified in the dermis (red arrows).

Day 14: Control group: complete re-epithelialization of the wound surface is observed. 0.9% NaCl group: partial coverage of the wound defect by a fibrinonecrotic scab persists (blue arrow). SVF, PRP, and PRP + SVF groups: the wound surface is completely re-epithelialized.

Day 30: Control group: skin appendages are absent or represented by isolated structures. 0.9% NaCl group: the dermis is poorly organized, and appendages are absent. SVF and PRP groups: partial restoration of hair follicles and sebaceous glands. PRP + SVF group: a large number of formed hair follicles and sebaceous glands with an organized structure comparable to intact skin are identified in the dermis.

Day 7: Control, 0.9% NaCl, and SVF groups: the dermis is characterized by persistence of a loose, immature collagenous matrix. PRP group: the dermis is represented by a loose disorganized collagen matrix with thin-walled vessels (red arrows). PRP + SVF group: the collagen matrix is represented by a loose matrix with dense fiber bundles (yellow arrow) and vessels (red arrows).

Day 14: Control group: the dermis shows a loose collagenous matrix. 0.9% NaCl group: the surface of the wound defect is covered with a dense layer of fibrinonecrotic material stained pink-red. The epithelial covering is absent. The underlying tissue is represented by a disorganized collagen matrix. SVF group: the extracellular matrix is composed of blue collagen fibers, among which denser, disorganized, intensely stained dark-blue collagen fibers are identified (yellow arrows). PRP group: the surface of the defect is completely covered by a continuous epithelial layer (black arrow). In the underlying tissue, thin evenly distributed collagen fibers stained in blue shades are observed. PRP + SVF group: the collagen matrix is represented by dense bundles of mature dark-blue fibers (yellow arrows). Areas of vascularization are identified (red arrows). The surface of the wound defect is completely covered by epithelium (black arrow).

Day 7: In the control group, the wounds appeared as a crater-like dermal defect filled with necrotic debris and fibrin. Beneath the scab, immature granulation tissue was identified, with moderate granulocytic infiltration (median score 1.5 [1; 2]) and a low density of newly formed capillaries (12 [10.75; 12.5] vessels/mm^2^). The thickness of the regenerative layer remained limited (median thickness 36.6% [25.6; 45.5]). The extracellular matrix was characterized by disorganization and a low density of thin collagen fibers (median score 3 [3; 3] for each parameter). In the 0.9% NaCl group, the wound bed contained a fibrinonecrotic scab without signs of epithelialization (Figure 4). Beneath it, granulation tissue with moderate granulocytic infiltration (median score 1 [1; 2]) and low vascularization (10.5 [9.75; 12.25] vessels/mm^2^) was observed. The regenerated layer was thin (median thickness 16% [13.5; 19.4]). The extracellular matrix was characterized by marked disorganization of collagen fibers and a predominance of thin collagen fibers (median score 3 [3; 3] for each parameter).

In the SVF group, the wound defect partially retained fibrinonecrotic material and was not completely epithelialized. The granulation tissue appeared more organized than in the control group and contained a greater number of newly formed capillaries (12 (9.75; 12.5) vessels/mm^2^). The thickness of the regenerative layer was moderate (median thickness 42.6% [39.2; 49.3]). The extracellular matrix demonstrated early signs of organization with initial fibroblast proliferation and was characterized by moderate disorganization (median score 2 [2; 3]) and a predominance of thin collagen fibers (median score 3 [2; 3]). In the PRP group, complete epithelialization of the wound defect was absent. The granulation tissue demonstrated a high density of newly formed vessels (22 (18.75; 26) vessels/mm^2^). The thickness of the regenerated layer reached 61% [54; 63.6]. The extracellular matrix was characterized by moderate disorganization (median score 2 [2; 2]) and a predominance of thin, immature collagen fibers (median score 2.5 [2; 3]). In the PRP + SVF group, re-epithelialization with formation of the spinous layer of the epidermis was observed. The granulation tissue was characterized by the highest density of newly formed vessels (24 (17.5; 35) vessels/mm^2^) and minimal inflammatory infiltration. The thickness of the regenerative layer reached the highest values among all groups (73% [69; 77.7]). The extracellular matrix was characterized by moderately disorganized collagen fibers (median score 2 [1.75; 2]) and a combination of immature collagen fibers and more organized mature collagen fibers (median score 2 [2; 3]).

Day 14: On day 14, in the control group, epithelialization remained incomplete (median epidermal differentiation score 1 [0.75; 1]). The granulation tissue was characterized by mild inflammatory infiltration and a small number of vessels (8 (6; 11) vessels/mm^2^). The thickness of the regenerative layer was 62.5% [51.3; 67.5]. In the 0.9% NaCl group, epithelialization from the wound edges was observed (median epidermal differentiation score 1 [0; 1]), while inflammatory infiltration and a low level of angiogenesis persisted (7.5 [6.5; 8.5] vessels/mm^2^). Focal necrosis persisted (median score 0.5 [0; 1]). The thickness of the regenerative layer remained minimal (56% [43.8; 63.8]).

In the SVF group, partial restoration of the epidermis was observed (1.5 [1; 2]). The granulation tissue was characterized by moderate vascularization (9 [7.5; 10] vessels/mm^2^). The thickness of the regenerative layer increased to 67% [61.8; 75.0]. In the PRP group, epidermal restoration with stratification of the spinous, granular, and keratinized layers was noted (median epidermal differentiation score 2 [2; 3]). The extracellular matrix contained a moderate density of vessels (14 [9; 16.5] vessels/mm^2^). Enhanced collagen deposition and further matrix organization were observed. The thickness of the regenerative layer reached 80% [73.8; 84.3]. In the PRP + SVF group, epidermal restoration and a high degree of epidermal differentiation were observed (median epidermal differentiation score 2 [2; 3]). The extracellular matrix contained a moderate density of vessels (15.5 [13.5; 19] vessels/mm^2^). The thickness of the regenerative layer reached the highest values (84.5% [80.5; 89.3]). Initial signs of skin appendage formation, including hair follicles, were also noted.

Day 30: In the control group, epithelialization of the wound defect was complete, while the degree of epidermal differentiation varied (median score 3 [2; 3]). The thickness of the regenerative layer reached 74% [64.8; 78.8]. The extracellular matrix was characterized by moderate organization of collagen fibers. In the 0.9% NaCl group, epithelialization with variable epidermal differentiation was observed (median score 3 [2; 3]). The thickness of the regenerative layer was minimal (median 65% [45.3; 72]). The extracellular matrix was poorly organized and was characterized predominantly by immature collagen fibers and isolated skin appendages.

In the SVF group, complete restoration of the epidermis was observed in the absence of inflammatory infiltration. The thickness of the regenerative layer reached 76% [68.8; 85]. The extracellular matrix demonstrated moderate organization with the presence of isolated hair follicles. In the PRP group, complete restoration of the epidermal structure with formation of a well-defined keratinized layer was observed (median epidermal differentiation score 3 [3; 3]). The thickness of the regenerated tissue was 82.5% [72.3; 89]. The dermis contained mature, dense collagen bundles and skin appendages. In the PRP + SVF group, complete restoration of the epidermis with differentiation of all its layers was noted. The thickness of the regenerative layer reached 87% [82.8; 93.3]. The extracellular matrix was characterized by the presence of a large number of well-organized mature collagen fibers. The histoarchitecture of the tissue most closely resembled that of intact skin outside the wound defect.

#### 3.2.2. Analysis of Wound Contraction and Closure Dynamics

Assessment of wound-healing dynamics using the indices proposed by Lemo [25] revealed marked intergroup differences in the kinetics of wound contraction and closure (Table 2).

Day 7: In the control group, a pronounced wound defect persisted (WSI = 0.634), with moderate values of SCI (0.146), DCI (0.366), and WCI (0.528). The negative GHI value (−0.080) indicates that, at this stage, the processes of contraction and granulation tissue formation were still insufficient to compensate for the initial injury. In the 0.9% NaCl group, high WSI values (0.688) were maintained, together with lower DCI (0.312) and WCI (0.444) values. Despite comparable SCI values (0.163), GHI remained negative (−0.244), indicating insufficient integration of superficial and deep regenerative processes.

In the SVF group, a decrease in WSI to 0.591 and an increase in DCI to 0.409 were accompanied by an increase in WCI to 0.564 and a shift in GHI into the positive range (0.018). These changes indicate activation of deep regenerative processes. In the PRP group, a more favorable reparative profile was observed. A marked decrease in WSI (to 0.420), together with a pronounced increase in DCI (0.580), resulted in an increase in WCI to 0.745 and GHI to 0.331. This pattern reflects active filling of the wound defect with granulation tissue and restoration of the dermal layer. At the same time, the relative stability of SCI confirms that the acceleration of healing was mainly due to enhanced deep regeneration rather than superficial contraction.

The most pronounced changes were recorded in the PRP + SVF group, which showed the lowest WSI (0.352) and the highest DCI (0.647), WCI (0.821), and GHI (0.457) values. This profile indicates the most intensive dermal restoration, rapid reduction in wound depth, and filling of the defect with regenerative tissue. Within the Lemo model, this corresponds to an optimal healing scenario in which deep contraction and dermal remodeling predominate, resulting in a regeneration-oriented healing pattern characterized by predominance of vertical tissue reconstruction over superficial contraction of the wound edges.

Day 14: On day 14, positive wound-healing dynamics were observed in all groups. In the control group, a decrease in WSI (to 0.375) and an increase in DCI (0.625) and WCI (0.771) were observed, while GHI remained positive (0.396), indicating a moderate rate of progressive healing with incomplete dermal restoration. In the 0.9% NaCl group, a positive value of the integrated GHI index (0.400), together with variability in WSI (0.340), DCI (0.660), and WCI (0.778), indicates uneven healing processes with alternating areas of more mature and less mature tissue.

In the SVF group, favorable dynamics were observed: a decrease in WSI to 0.330 and an increase in DCI to 0.670 were accompanied by an increase in WCI to 0.829 and GHI to 0.484, reflecting partial filling of the wound defect. In the PRP group, an acceleration of repair was noted. A decrease in WSI to 0.200, together with a marked increase in DCI to 0.800 and high values of WCI (0.888) and GHI (0.670), indicates nearly complete wound closure and active dermal restoration. The most pronounced changes persisted in the PRP + SVF group. The lowest WSI (0.155) and the highest DCI (0.845) were accompanied by the highest WCI (0.957) and GHI (0.808) values, indicating healing of the wound defect and restoration of the dermis. In the Lemo model, this profile corresponds to an optimal healing scenario in which, after completion of epithelialization, deep contraction and tissue remodeling processes predominate.

Day 30: In the control group, a further decrease in WSI (to 0.260) and an increase in DCI (0.740) and WCI (0.878) were observed. The GHI reached 0.623, reflecting nearly complete regeneration of the wound defect. The remodeling indices remained moderate, which, together with the MMRI value (0.404), indicates immaturity of the newly formed dermis and ongoing remodeling of the extracellular matrix. In the 0.9% NaCl group, despite comparable wound closure indices—WSI (0.255), DCI (0.745), WCI (0.861), and GHI (0.645)—the MMRI value (0.343) was lower than in the other groups, reflecting only moderate remodeling.

In the SVF group, satisfactory healing dynamics were observed: WSI decreased to 0.240, DCI increased to 0.760, and WCI reached 0.910 with a GHI of 0.675, indicating completion of wound closure and dermal formation. Moderate MMRI values (0.417) indicate limited remodeling activity. In the PRP group, a decrease in WSI to 0.175 and an increase in DCI to 0.825 were accompanied by high WCI (0.961) and GHI (0.796) values. The increase in MMRI to 0.502 indicates active tissue remodeling. In the PRP + SVF group, the lowest WSI (0.130) and highest DCI (0.870) were combined with the highest WCI (0.973) and GHI (0.843) values, indicating near-complete restoration of the dermal layer. The highest MMRI value (0.655) indicates the formation of mature, structurally organized tissue. Within the Lemo model, this corresponds to the final stage of healing, in which deep contraction and remodeling fully compensate for the original defect.

## 4. Discussion

In the present study, we evaluated the regenerative effects of platelet-rich plasma (PRP) and stromal vascular fraction (SVF), applied both individually and in combination 24 h before wound creation in a rabbit full-thickness excisional skin wound model. Preoperative intradermal administration of PRP, either alone or in combination with SVF, was associated with more pronounced epidermal differentiation on days 7 and 14 compared with the untreated control and saline-treated groups.

The most pronounced intergroup differences were observed during the proliferative phase on days 7 and 14, indicating an accelerated transition from the inflammatory to the reparative stage of healing. As early as day 7, the PRP group, and especially the PRP + SVF group, showed an earlier onset of re-epithelialization with the formation of a multilayered epidermis, whereas epithelialization was absent in the control groups. By day 14, these differences persisted and became more pronounced, accompanied by wound closure with a more differentiated epidermis and the development of granular and cornified layers. In contrast, the isolated use of SVF did not result in a comparable acceleration of epithelialization.

The observed dynamics are consistent with current concepts regarding the mechanisms of PRP action, according to which its high concentration of growth factors stimulates keratinocyte proliferation and migration, thereby accelerating restoration of the epidermal layer [26,27,28,29]. At the same time, the acceleration of epithelialization may be associated not only with the direct effects of growth factors, but also with modulation of the inflammatory phase. In the PRP and PRP + SVF groups, a more rapid reduction in the severity of inflammatory infiltration was observed, manifested by a decrease in the number of granulocytes already at the early observation time points compared with the control groups. This is consistent with published data showing that PRP contributes to regulation of the cytokine profile and activation of reparative macrophages, thereby creating favorable conditions for keratinocyte migration [30,31,32]. In turn, the cellular component of SVF, which includes mesenchymal stromal cells, likely enhances this effect through paracrine secretion of biologically active factors that promote keratinocyte activation and accelerate epidermal restoration [33,34,35,36]. However, the cytokine profile and keratinocyte proliferation were not assessed in the present study; therefore, these proposed mechanisms remain hypothetical.

SVF contains mesenchymal stromal cells, endothelial progenitors, pericytes, and immunomodulatory cells capable of sustained paracrine signaling [6,37,38]. Through secretion of VEGF, HGF, SDF-1, and anti-inflammatory cytokines, SVF promotes angiogenesis, fibroblast activation, and extracellular matrix remodeling [38,39]. Activation of signaling axes such as SDF-1/CXCR4 and STAT3 likely enhances endothelial recruitment and stabilizes neovascular networks [40,41]. Moreover, SVF-derived mesenchymal cells may facilitate macrophage polarization toward a reparative M2 phenotype, promoting transition from inflammation to proliferation [42,43].

The regenerative effect observed in the PRP + SVF group may be explained by the combined biological activity of PRP-derived growth factors and the cellular component of SVF. Similar effects were demonstrated in the experimental study by Griffeth et al. [44], which showed enhancement of the regenerative potential of MSCs under the influence of PRP. The obtained findings are also consistent with reports from other authors [45,46] showing that the combined use of mesenchymal stromal cells and PRP is associated with accelerated wound healing compared with monotherapy and control conditions. In contrast to these studies, the present work evaluated the effect of preoperative administration of PRP and SVF 24 h before wound creation. Our findings suggest that this regimen is associated with enhanced reparative processes. Nevertheless, the study design does not allow us to conclusively determine whether the observed effects resulted from biological tissue preconditioning or from the sustained local biological activity of the administered products. Therefore, although preoperative administration of PRP and SVF may contribute to the establishment of a tissue microenvironment more favorable for subsequent wound healing, further studies are required to confirm this hypothesis.

Our findings are consistent with those reported by Li Z et al. [47]. In contrast to our study, in which PRP and SVF were administered before the creation of a full-thickness excisional wound, Li Z et al. applied the combined intervention after the induction of radiation-induced skin injury in rabbits. In their study, administration of SVFG and APRFE was associated with accelerated wound healing and increased collagen deposition. Despite differences in the injury models, timing of intervention, and evaluated outcomes, the findings of both studies support the regenerative and pro-angiogenic potential of combining SVF-based preparations with platelet-derived products.

Additional evidence supporting the regenerative potential of platelet-derived bioproducts was provided by Jia L. et al. [48], who reported that the use of concentrated growth factor as part of combination therapy was associated with hair regrowth in a clinical model of alopecia areata. Although direct comparison with our experimental model is limited by differences in study design and the nature of the underlying pathology, the findings of Jia L. et al. [48] are consistent with our results regarding the regenerative potential of platelet-derived bioproducts.

Quantitative assessment of ERG-positive microvessel density demonstrated an increased number of vessels in the PRP and PRP + SVF groups compared with the control groups. On day 7, the median microvessel density was significantly higher in the PRP and PRP + SVF groups, reaching 22 [18.75; 26] vessels/mm^2^ and 24 [17.5; 35] vessels/mm^2^, respectively, compared with the untreated control, 0.9% NaCl, and SVF groups. By day 14, microvessel density remained higher in the PRP and PRP + SVF groups, reaching 14 [9; 16.5] and 15.5 [13.5; 19] vessels/mm^2^, respectively. Although the overall intergroup differences remained statistically significant, a significant pairwise difference was observed only between the 0.9% NaCl and PRP + SVF groups (Bonferroni-adjusted *p* = 0.016). By day 30, no differences in the number of newly formed vessels were observed between the groups, which likely reflects completion of the active phase of neovascularization and transition to remodeling.

Biologically, this effect may be explained by the high content of proangiogenic growth factors in PRP, including VEGF, PDGF, FGF, and TGF-β which stimulate endothelial cell proliferation and migration, capillary formation, and maturation of granulation tissue, in agreement with the published literature [31,32]. The additional contribution of SVF in the PRP + SVF group may be related to the presence of endothelial progenitor cells, pericytes, and mesenchymal stromal cells capable of supporting vascular regeneration through paracrine secretion of VEGF, HGF, SDF-1, and extracellular vesicles. These mechanisms promote endothelial cell migration, vascular tube formation, and stabilization of newly formed vessels [33,49].

The greater number of vessels in the PRP + SVF group compared with SVF monotherapy indicates that isolated administration of the cellular fraction without a sufficient amount of exogenous growth factors may be insufficient to rapidly initiate neovascularization in the setting of an acute wound. PRP likely provides early stimulation, whereas SVF supports and prolongs the regenerative response through cell-mediated paracrine mechanisms. At the same time, the absence of significant differences by day 30 emphasizes that the main advantage of combined therapy lies not in prolonged stimulation of vascular growth, but in earlier activation of the angiogenic phase of healing.

One of the key differences between the study groups was the remodeling of the extracellular matrix at the later stages of healing. As early as day 14, maturation and improved organization of the extracellular matrix were observed in the PRP and especially the PRP + SVF groups compared with the control groups, accompanied by a trend toward increased thickness of the regenerative layer (up to 80% and 84.5%, respectively, versus 62.5% in the control group). In the PRP + SVF group, the matrix was characterized by a higher degree of structural organization and the formation of skin appendages. By day 30, these differences became more pronounced: the PRP + SVF group showed the highest regenerative layer thickness values (87% [82.8; 93.3]) and the greatest degree of collagen fiber maturity, whereas the control groups retained a thinner and less organized dermis. The matrix remodeling index in the PRP + SVF group also reached the highest values, indicating the formation of mature and functionally organized tissue resembling intact skin.

Importantly, by day 30, the PRP + SVF group also demonstrated more pronounced restoration of skin appendages compared with the control groups (median 3 [2.75; 3] versus 1 [1; 2], Bonferroni-adjusted *p* = 0.005), indicating more complete recovery of skin structure and function. The obtained findings are consistent with studies demonstrating a higher density of collagen fibers following wound treatment with PRP and mesenchymal stem cells [45,50]. The more pronounced effect observed in the PRP + SVF group may reflect a complementary interaction between PRP-derived growth factors and SVF-derived cellular components, whereby PRP contributes to the initiation of early extracellular matrix formation, while SVF supports its subsequent maturation and structural organization. In turn, preoperative administration may contribute to preliminary activation of the tissue microenvironment, thereby promoting more effective restoration of skin architecture and reducing the risk of pathological scarring.

Importantly, in the SVF monotherapy group, despite an increase in regenerative layer thickness, lower collagen matrix maturity was observed by day 30, indicating that the cellular component alone may be insufficient for effective remodeling in the absence of the early stimulatory influence of growth factors.

The inclusion of a physiological saline (NaCl) group made it possible to minimize the potential influence of the injection procedure itself on the healing process. The absence of statistically significant differences between the control and NaCl groups indicates that the observed effects were not related to mechanical tissue injury caused by injection, but rather were attributable to the biological action of PRP and its combination with SVF.

The findings may indicate complementary biological activity of PRP and SVF. PRP may provide an early reservoir of growth factors that facilitates the transition from the inflammatory to the proliferative phase of wound healing, whereas SVF may contribute to later reparative processes through its heterogeneous cellular composition and sustained paracrine activity. Notably, while the PRP + SVF group showed significant superiority during the early and mid-stages (Days 7–14), by Day 30, the regenerative thickness was comparable to the SVF monotherapy group, suggesting that SVF plays a dominant role in long-term structural maturation. Further studies are needed to clarify the contribution of each component to early wound healing and subsequent tissue remodeling.

The physiological significance of the findings observed on Day 7 may lie in their indication of an earlier activation of regenerative processes after preoperative administration of PRP and SVF. By accelerating the initial transition from inflammation to the early proliferative phase, the PRP + SVF conditioning creates a robust scaffold that prevents wound stagnation. This early regenerative stimulus may facilitate subsequent tissue remodeling and the restoration of skin appendages observed during the later stages of the study.

Crucially, the early restoration of skin appendages observed by Day 14 in the combined group underscores the superior regenerative potential of this additive strategy. While PRP promotes rapid cell migration, it is the cellular diversity of the SVF—particularly its mesenchymal and endothelial progenitor populations—that likely provides the necessary niche for adnexal organogenesis. These cells facilitate the complex epithelial–mesenchymal interactions required for hair follicle and gland bud formation, a process typically absent in standard wound repair or PRP monotherapy. This bidirectional interaction may explain the earlier epithelial differentiation, more organized collagen type I deposition, and improved architectural restoration observed in the combined group at days 14 and 30. Such findings suggest not merely accelerated healing but improved architectural restoration.

Previous studies have demonstrated beneficial effects of PRP and SVF in chronic wounds and experimental models [36,37,38]; however, these interventions were consistently applied after wound creation (post-injury or topical application) [45,46,47]. To the best of our knowledge, published data regarding the preoperative administration of PRP and SVF prior to surgical skin injury remain limited. Preoperative biological tissue conditioning may represent a promising strategy for enhancing subsequent wound healing; however, whether the observed effects specifically reflect the contribution of preoperative tissue conditioning remains unclear.

Nevertheless, despite the widespread use of the rabbit full-thickness wound model in cutaneous regeneration studies, wound healing mechanisms in rabbits differ substantially from those in humans. In rabbits, in contrast to humans, wound closure is largely driven by contraction due to the presence of the panniculus carnosus. Moreover, interspecies differences in skin structure, immune response, and tissue biomechanics may affect the observed regenerative effects of PRP and SVF. Therefore, further validation in large-animal models and clinical studies is required before these findings can be translated to human wound healing.

This study has several limitations that should be taken into account when interpreting the results. The present study was designed as a pilot investigation; therefore, a formal sample size calculation was not performed. The relatively small sample size reduces statistical power and may limit the reproducibility of the findings. The present study did not include a comparative evaluation of different PRP and SVF doses or administration intervals, nor did it include a postoperative treatment group. Therefore, the findings should be considered preliminary and require confirmation in prospective experimental studies with adequate statistical power. This limits our ability to distinguish the relative contribution of biological tissue preconditioning from the sustained local biological activity of the administered products to the observed regenerative effects. In addition, SVF characterization was limited to flow cytometric assessment of the CD34+/CD45− cell population, without multiparametric phenotyping. Because mechanically isolated SVF may differ from enzymatically derived SVF in terms of cellular composition and the distribution of individual cell subpopulations, extrapolation of the present findings to SVF obtained using other isolation methods should be approached with caution. The observation period made it possible to assess the early stages of repair; however, it was insufficient for the evaluation of the final maturation of scar tissue and its functional integrity. A limitation of this study is the absence of molecular investigations, including cytokine profiling and gene expression analysis. As a result, the interpretation of the mechanisms underlying the reparative potential of PRP and SVF is based primarily on our morphological findings and previously published data. Furthermore, functional assessment of the regenerated tissue, including biomechanical strength, elasticity, and scar quality, was not performed. Nevertheless, these findings expand current understanding of preoperative skin conditioning with PRP + SVF and support further investigation of the biological relevance of PRP and SVF in regenerative medicine and plastic surgery.

## 5. Conclusions

Preoperative intradermal administration of PRP combined with SVF 24 h before wound creation enhanced the reparative response of postoperative wounds compared with both the control group and PRP monotherapy. These findings suggest that preoperative intradermal administration of PRP combined with SVF may represent a promising strategy to enhance the histological and histomorphometric outcomes of surgical wound repair. However, the present study does not allow us to distinguish between the effects of biological tissue preconditioning and the persistent local biological activity of the administered preparations. These preliminary findings require further adequately powered preclinical studies incorporating postoperative administration groups, different preoperative administration intervals, standardized treatment protocols, and assessment of their potential clinical relevance.

## Figures and Tables

**Table 1:** Semi-quantitative scoring system used for histological evaluation of wound healing parameters.

Parameter	0	1	2	3
Epidermal differentiation	Absent	Formation of a spinous layer	Presence of a granular layer with keratohyalin granules	Complete keratinization with formation of a stratum corneum
Granulocytes (inflammatory infiltrate)	Absent	Isolated cells	Moderate infiltration	Dense infiltration
Lymphocytes (inflammatory infiltrate)	Absent	Isolated cells	Moderate infiltration	Dense infiltration
Necrosis	Absent	Mild (detected in 1–3 of 9 fields of view)	Moderate (detected in 4–6 of 9 fields of view)	Severe (detected in 7–9 of 9 fields of view)
Granulation tissue	Absent	Scant	Moderate	Abundant
Granulation tissue maturity	Mature	Moderate degree of maturity	Low degree of maturity	Immature
Organization of collagen fibers	Comparable to intact dermis	Mild disorganization	Moderate disorganization	Marked disorganization
Collagen fiber density	High	Moderately high	Moderately low	Low
Collagen fiber thickness	Predominantly thick bundles	Mixed, with a predominance of thick bundles	Mixed, with a predominance of thin bundles	Predominantly thin bundles
Fibrosis	Absent	Mild	Moderate	Marked
Skin appendages (hair follicles and sebaceous glands)	Absent	Isolated structures	Partial regeneration	Complete restoration

**Table 2:** Calculation of wound-healing indices at each experimental time point.

Time Point	Groups	WSI (Wound Severity Index)	SCI (Superficial Contraction Index)	DCI (Deep Contraction Index)	WCI (Wound Contraction Index)	GHI (Global Healing Index)	MMRI (Modified Matrix Remodelling Index)
Day 7	Control (1)	0.634 (0.545–0.744)	0.146 (0.104–0.177)	0.366 (0.256–0.455)	0.528 (0.460–0.628)	−0.080	-
	0.9% NaCl (2)	0.688 (0.662–0.717)	0.163 (0.006–0.258)	0.312 (0.283–0.338)	0.444 (0.396–0.521)	−0.244	-
	SVF (3)	0.591 (0.543–0.608)	0.155 (0.082–0.183)	0.409 (0.392–0.457)	0.564 (0.488–0.638)	0.018	-
	PRP (4)	0.420 (0.398–0.440)	0.155 (0.115–0.209)	0.580 (0.560–0.602)	0.745 (0.675–0.830)	0.331	-
	PRP + SVF (5)	0.352 (0.321–0.384)	0.158 (0.073–0.205)	0.647 (0.615–0.679)	0.821 (0.776–0.856)	0.457	-
	*p*-value	*p*_0_ < 0.0001 *p*_1–5_ = 0.004 *p*_2–4_ = 0.004 *p*_2–5_ < 0.0001 *p*_3–5_ = 0.047	*p*_0_ = 0.998	*p*_0_ < 0.0001 *p*_1–5_ = 0.004 *p*_2–4_ = 0.004 *p*_2–5_ < 0.0001 *p*_3–5_ = 0.047	*p*_0_ < 0.0001 *p*_1–5_ = 0.010 *p*_2–4_ = 0.006 *p*_2–5_ < 0.0001 *p*_3–5_ = 0.033	*p*_0_ < 0.0001 *p*_1–5_ = 0.003 *p*_2–4_ = 0.003 *p*_2–5_ < 0.0001 *p*_3–5_ = 0.027	
Day 14	Control (1)	0.375 (0.325–0.488)	0.134 (0.101–0.177)	0.625 (0.513–0.675)	0.771 (0.704–0.836)	0.396	0.318 (0.200–0.363)
	0.9% NaCl (2)	0.340 (0.261–0.562)	0.192 (0.006–0.258)	0.660 (0.437–0.739)	0.778 (0.615–0.858)	0.400	0.283 (0.198–0.370)
	SVF (3)	0.330 (0.250–0.383)	0.185 (0.147–0.284)	0.670 (0.618–0.750)	0.829 (0.792–1.024)	0.484	0.333 (0.241–0.350)
	PRP (4)	0.200 (0.158–0.263)	0.140 (0.115–0.154)	0.800 (0.738–0.843)	0.888 (0.856–0.983)	0.670	0.414 (0.379–0.454)
	PRP + SVF (5)	0.155 (0.108–0.195)	0.109 (0.055–0.122)	0.845 (0.805–0.893)	0.957 (0.946–0.967)	0.808	0.556 (0.400–0.575)
	*p*-value	*p*_0_ = 0.005 *p*_1–5_ = 0.018 *p*_2–5_ = 0.024	*p*_0_ = 0.215	*p*_0_ = 0.005 *p*_1–5_ = 0.018 *p*_2–5_ = 0.024	*p*_0_ = 0.208	*p*_0_ = 0.045	*p*_0_ = 0.017 *p*_1–5_ = 0.007 *p*_2–5_ = 0.008
Day 30	Control (1)	0.260 (0.213–0.353)	0.146 (0.104–0.188)	0.740 (0.648–0.788)	0.878 (0.779–0.966)	0.623	0.404 (0.348–0.478)
	0.9% NaCl (2)	0.255 (0.175–0.547)	0.167 (0.128–0.194)	0.745 (0.453–0.825)	0.861 (0.662–0.954)	0.645	0.343 (0.190–0.418)
	SVF (3)	0.240 (0.150–0.313)	0.134 (0.063–0.149)	0.760 (0.688–0.850)	0.910(0.786–0.975)	0.675	0.417 (0.300–0.437)
	PRP (4)	0.175 (0.110–0.278)	0.124 (0.078–0.188)	0.825 (0.723–0.890)	0.961 (0.852–1.000)	0.796	0.502 (0.446–0.591)
	PRP + SVF (5)	0.130 (0.068–0.173)	0.077 (0.060–0.115)	0.870 (0.828–0.933)	0.973 (0.895–1.000)	0.843	0.655 (0.630–0.771)
	*p*-value	*p*_0_ = 0.078	*p*_0_ = 0.228	*p*_0_ = 0.078	*p*_0_ = 0.341	*p*_0_ = 0.161	*p*_0_ = 0.017 *p*_1–5_ = 0.001 *p*_2–5_ < 0.0001

Note: Data are presented as Median (Interquartile Range: 25th–75th percentiles). Statistical analysis performed via Kruskal–Wallis test followed by Dunn’s post hoc with Bonferroni correction. *p* is the significance level; *p*_0_—comparison of all groups using the Kruskal–Wallis test; *p*_1–2_—Control vs. NaCl; *p*_1–3_—Control vs. SVF; *p*_1–4_—Control vs. PRP; *p*_1–5_—Control vs. PRP + SVF; *p*_2–3_—NaCl vs. SVF; *p*_2–4_—NaCl vs. PRP; *p*_2–5_—NaCl vs. PRP + SVF; *p*_3–4_—SVF vs. PRP; *p*_3–5_—SVF vs. PRP + SVF; *p*_4–5_—PRP vs. PRP + SVF.

## Data Availability

The original contributions presented in this study are included in the Supplementary Materials. Further inquiries can be directed to the corresponding author.

## Supplementary Materials

The following supporting information can be downloaded at: https://www.mdpi.com/article/10.3390/biomedicines14102399/s1, Table S1: (A) Histopathological and morphometric evaluation of wound healing after 7 days. (B) Histopathological and morphometric evaluation of wound healing after 14 days. (C) Histopathological and morphometric evaluation of wound healing after 30 days.
