# Supplementary material for: Preoperative Skin Preconditioning with Autologous PRP and SVF: Effects on Cutaneous and Adnexal Repair in a Rabbit Pilot Study
Source: Biomedicines. 2026 Oct 20;14(10):2399. doi: 10.3390/biomedicines14102399 (PMC13702528; doi:10.3390/biomedicines14102399)
Supplement: Supplementary file 1 [file biomedicines-14-02399-s001.zip › biomedicines-4621799-supplementary.pdf]

Table S1. (A) Histopathological and morphometric evaluation of wound healing after 7 days. (B) Histopathological and morphometric evaluation of wound healing after 14 days. (C) Histopathological and morphometric evaluation of wound healing after 30 days

**(A)**						
**Parameter**	**Control** (1) n = 8	**0.9% NaCl** (2) n = 8	**SVF** (3) n = 8	**PRP** (4) n = 8	**PRP + SVF** (5) n = 8	**p-value**
Differentiation of epidermis, score (0-3)	0 (0; 0)	0 (0; 0)	0 (0; 0)	1 (0,75; 2)	1,5 (1; 2)	p_0 **< 0,0001** p_{1-4} = **0,024**, p_{1-5} = **0,003**, p_{2-4} = **0,024**, p_{2-5} = **0,003**, p_{3-5} = **0,013**
Microvessel density, vessels/mm²	12 (10,75; 12,5)	10,5 (9,75; 12,25)	12 (9,75; 12,5)	22 (18,75; 26)	24 (17,5; 35)	p_0 **< 0,0001** p_{1-4} = **0,025**, p_{1-5} = **0,027**, p_{2-4} = **0,003**, p_{2-5} = **0,003**, p_{3-4} = **0,012**, p_{3-5} = **0,013**
Granulocytes, score (0-3)	1,5 (1; 2)	1 (1; 2)	1 (1; 1,25)	1 (0; 1,25)	0 (0; 1)	p_0 = **0,019** p_{1-5} = **0,035**, p_{2-5} = **0,019**
Lymphocytes, score (0-3)	2 (1,75; 2,25)	2 (1; 2)	1 (1; 2)	1 (1; 1,25)	1 (1; 1,25)	p_0 = 0,095
Necrosis, score (0-3)	1 (0,75; 1)	1,5 (0; 2)	1 (0; 1)	0 (0; 1)	0 (0;0)	p_0 = 0,063
Granulation tissue, score (0-3)	2 (2; 3)	2 (2; 2.25)	3 (2; 3)	3 (3; 3)	3 (3; 3)	p_0 = **0,002** p_{2-4} = **0,014**, p_{2-5} = **0,014**
Granulation tissue maturity, score (0-3)	3 (2,75; 3)	3 (3; 3)	3 (2; 3)	2 (2; 3)	2 (2; 2)	p_0 = **0,016** p_{2-5} = **0,020**
Collagen fiber density, score (0-3)						
Collagen organization	3 (3; 3)	3 (3; 3)	2 (2; 3)	2 (2; 2)	2 (1,75; 2)	p_0 **< 0,0001** p_{1-4} = **0,008**, p_{1-5} = **0,003**, p_{2-4} = **0,008**, p_{2-5} = **0,016**
Density	3 (3; 3)	3 (3; 3)	3 (2; 3)	3 (2; 3)	2,5 (2; 3)	p_0 = 0,158
Thickness	3 (3; 3)	3 (3; 3)	3 (2; 3)	2,5 (2; 3)	2 (2; 3)	p_0 = 0,043
Dermal fibrosis, score (0-3)	0 (0; 0)	0 (0;0)	0 (0; 0)	0 (0;0)	0 (0;0)	p_0 = 1,00
Regenerative thickness (%)	36,6 (25,6; 45,5)	16 (13,5; 19,4)	42,6 (39.2; 49,3)	61 (54; 63,6)	73 (69; 77,7)	p_0 **< 0,0001** p_{1-5} = **0,005**, p_{2-4} = **0,001**, p_{2-5} **< 0,0001**, p_{3-5} = **0,033**
Skin appendages, score (0-3)	0 (0;0)	0 (0;0)	0 (0;0)	0 (0;0)	0 (0; 0)	p_0 = 1,00
**(B)**						
**Parameter**	**Control** (1) n = 8	**0.9% NaCl** (2) n = 8	**SVF** (3) n = 8	**PRP** (4) n = 8	**PRP + SVF** (5) n = 8	**p-value**
Differentiation of epidermis, score (0-3)	1 (0,75; 1)	1 (0; 1)	1,5 (1; 2)	2 (2; 3)	2 (2; 3)	p_0 **< 0,0001** p_{1-4} = **0,009**, p_{1-5} = **0,009**, p_{2-4} = **0,001**, p_{2-5} = **0,001**
Microvessel density, vessels/mm²	8 (6; 11)	7,5 (6,5; 8,5)	9 (7,5; 10)	14 (9; 16,5)	15,5 (13,5; 19)	p_0 = **0,004** p_{2-5} = **0,016**
Granulocytes, score (0-3)	0,5 (0; 1)	1 (0; 1)	0 (0; 0)	0 (0; 0)	0 (0; 0)	p_0 = 0,066
Lymphocytes, score (0-3)	1 (1; 1)	1 (0,75; 1)	1 (0; 1)	0,5 (0; 1)	0,5 (0; 1)	p_0 = 0,302
Necrosis, score (0-3)	0 (0; 0)	0,5 (0; 1)	0 (0;0)	0 (0;0)	0 (0;0)	p_0 = **0,002** p_{1-2} = **0,010**, p_{2-3} = **0,010**, p_{2-4} = **0,010**, p_{2-5} = **0,010**
Granulation tissue, score (0-3)	2 (1; 2)	1,5 (1; 2)	2 (2; 2)	2 (1; 2)	1 (1; 2)	p_0 = 0,303
Granulation tissue maturity, score (0-3)	2 (1,75; 2)	2 (2; 3)	2 (1; 2,25)	1 (1; 2)	0,5 (0; 1)	p_0 = **0,001** p_{2-5} = **0,001**
Collagen fiber density,						

score (0-3)						
Collagen organization	2 (2; 2)	3 (3; 3)	2 (1,75; 2)	2 (2; 2)	1,5 (1; 2)	p_0 < **0,0001**<br>p_{1-2} = **0,020**, p_{2-3} = **0,001**, p_{2-5} < **0,0001**
Density	2 (2; 2,25)	3 (3; 3)	2 (1,75; 2)	2 (2; 2)	2 (1; 2)	p_0 = **0,0001**<br>p_{2-3} = **0,005**, p_{2-5} = **0,001**
Thickness	2 (2; 2)	3 (3; 3)	2 (1; 2)	1,5 (1; 2)	2 (1; 2)	p_0 **< 0,0001**<br>p_{2-3} = **0,002**, p_{2-4} **< 0,0001**, p_{2-5} = **0,002**
Dermal fibrosis, score (0-3)	1 (1; 1)	1 (0,75; 2)	1 (1; 1)	1 (1; 1)	1 (0; 1)	p_0 = 0,622
Regenerative thickness (%)	62,5<br>(51,3; 67.5)	56.0<br>(43,8; 63,8)	67<br>(61,8; 75,0)	80<br>(73,8; 84.3)	84,5<br>(80,5; 89,3)	p_0 = **0,0001**<br>p_{1-5} = **0,021**, p_{2-4} = **0,049**, p_{2-5} = **0,001**
Skin appendages, score (0-3)	0 (0; 0)	0 (0; 0)	0 (0; 0,25)	0 (0; 0)	1 (0; 1)	p_0 = **0,003**<br>p_{1-5} = **0,012**, p_{2-5} = **0,012**, p_{4-5} = **0,012**
**(C)**						
**Parameter**	**Control**<br>(1)<br>n = 8	**0.9% NaCl**<br>(2)<br>n = 8	**SVF**<br>(3)<br>n = 8	**PRP**<br>(4)<br>n = 8	**PRP + SVF**<br>(5)<br>n = 8	**p-value**
Differentiation of epidermis, score (0-3)	3 (2; 3)	3 (2; 3)	3 (3; 3)	3 (3; 3)	3 (3; 3)	p_0 = 0,098
Microvessel density, vessels/mm²	9 (8,25; 10,5)	9 (7,5; 10,25)	10 (8; 11)	10 (8; 16)	11,5 (8; 16,5)	p_0 = 0,708
Granulocytes, score (0-3)	0 (0; 0)	0 (0; 0)	0 (0; 0)	0 (0; 0)	0 (0; 0)	p_0 = 0,545
Lymphocytes, score (0-3)	0 (0; 0)	0 (0; 0)	0 (0; 0)	0 (0; 0)	0 (0; 0)	p_0 = 0,406
Некроз, score (0-3)	0 (0; 0)	0 (0; 0)	0 (0; 0)	0 (0; 0)	0 (0; 0)	p_0 = 0,406
Granulation tissue maturity, score (0-3)	0 (0; 0)	0 (0; 0)	0 (0; 0)	0 (0; 0)	0 (0; 0)	p_0 = 0,406
Collagen fiber density,<br>score (0-3)						
Collagen organization	2 (1; 2)	2 (1; 2)	1 (1; 2)	2 (1; 2)	1 (1; 1)	p_0 = 0,115
Density	2 (1; 2)	2 (1; 2)	1 (1; 2)	2 (1; 2)	1 (1; 2)	p_0 = 0,536
Thickness	2 (1; 2)	1 (1; 2)	1 (1; 2)	1 (1; 2)	1 (1; 2)	p_0 = 0,844
Dermal fibrosis, score (0-3)	1 (1; 1,25)	1 (0,75; 2)	1 (1; 1)	1 (1; 1)	1 (0; 1)	p_0 = 0,395
Regenerative thickness (%)	74<br>(64,8; 78,8)	65<br>(45,3; 72)	76<br>(68,8; 85)	82,5<br>(72,3; 89)	87<br>(82.8; 93,3)	p_0 = 0,072
Skin appendages, score (0-3)	1 (1; 2)	1 (0.75; 2)	2 (1; 2)	2 (1; 2)	3 (2.75; 3)	p_0 = **0,001**<br>p_{1-5} = **0,005**, p_{2-5} = **0,001**

Note: Data are presented as Median (Interquartile Range: 25th–75th percen-tiles). Sta-tistical analysis performed via Kruskal-Wallis test followed by Dunn's post-hoc with Bonferroni correction. p is the significance level; p0 – comparison of all groups using the Kruskal–Wallis test. All p-values for pairwise comparisons (from p1–2 to p4–5) were adjusted using the Bonferroni method. p1-2 - Control vs NaCl; p1-3 - Control vs SVF; p1-4 - Control vs PRP; p1-5 - Control vs PRP+SVF; p2-3 - NaCl vs SVF; p2-4 - NaCl vs PRP; p2-5 - NaCl vs PRP+SVF; p3-4 - SVF vs PRP; p3-5 - SVF vs PRP+SVF; p4-5 - PRP vs PRP+SVF.

[a] – The number of microvessels was determined by counting ERG-positive microvessels per mm².
